# Supplementary material for: High-Loaded Copper-Containing Sol–Gel Catalysts for Furfural Hydroconversion
Source: Int J Mol Sci. 2023 Apr 19;24(8):7547. doi: 10.3390/ijms24087547 (PMC10142956; doi:10.3390/ijms24087547)
Supplement: Supplementary file 1 [file ijms-24-07547-s001.zip › ijms-2311846-supplementary.pdf]

Article

High-Loaded Copper-Containing Sol-Gel Catalysts for Furfural Hydroconversion

Svetlana Selishcheva*, Anastasiya Sumina, Evgeny Gerasimov, Dmitry Selishchev, and Vadim Yakovlev

Boreskov Institute of Catalysis, Lavrentiev Ave. 5, Novosibirsk 630090, Russia; sumina@catalysis.ru (A.S.); gerasimov@catalysis.ru (E.G.); selishev@catalysis.ru (D.S.); yakovlev@catalysis.ru (V.Y.)

* Correspondence: svetlana@catalysis.ru; Tel.: +7(383)326-96-67

Supplementary

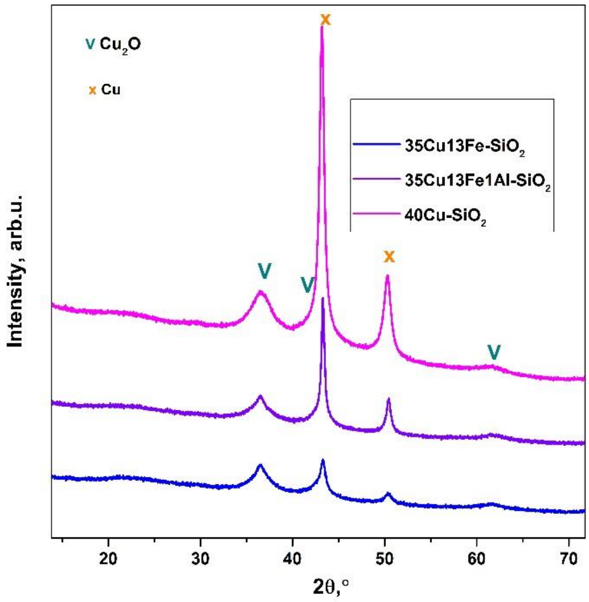

Figure S1. XRD patterns of the copper-containing catalysts after reaction.

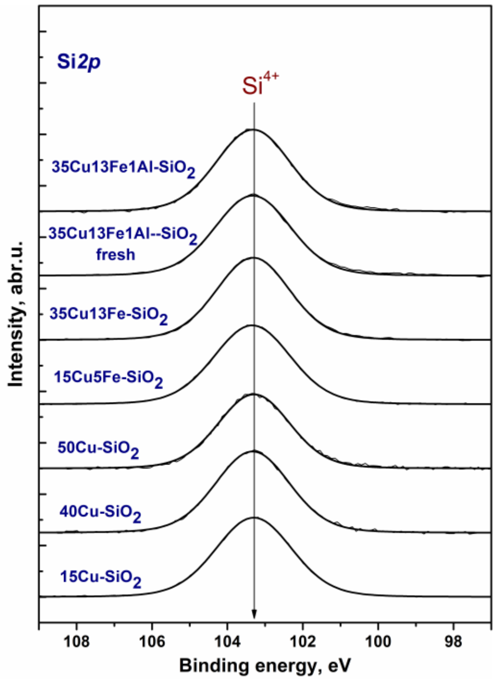

Figure S2. Si2p spectra of the Cu-containing catalysts.

Table S1. Binding energies of Si2p, C1s, Cu2p$_{3/2}$, Fe2p$_{3/2}$ и O1s (eV).

Catalyst	Si2p	Cu2p$_{3/2}$	Auger parameter	Fe2p$_{3/2}$	O1s	C1s
15Cu-SiO$_2$	103.3	932.5<br>935.1	–<br>–		532.6	284.6
40Cu-SiO$_2$	103.3	932.5<br>935.2	1849.3<br>1851.2		530.7 (CuO$_x$)<br>532.6	284.7
50Cu-SiO$_2$	103.3	932.5<br>935.1	1849.2<br>1851.2		530.7 (CuO$_x$)<br>532.6	284.8
15Cu5Fe-SiO$_2$	103.3	932.5<br>935.0	–<br>–	711.6	532.6	284.8
35Cu13Fe-SiO$_2$	103.3	932.5<br>935.2	1849.0<br>1851.2	712.1	530.7 (CuO$_x$)<br>532.6	284.8
35Cu13Fe1Al-SiO$_2$ (fresh)	103.3	935.1	1851.3	711.6	530.7 (CuO$_x$)<br>532.6	284.8
35Cu13Fe1Al-SiO$_2$	103.3	932.5<br>935.2	1849.1<br>1851.2	712.3	530.7 (CuO$_x$)<br>532.6	284.9

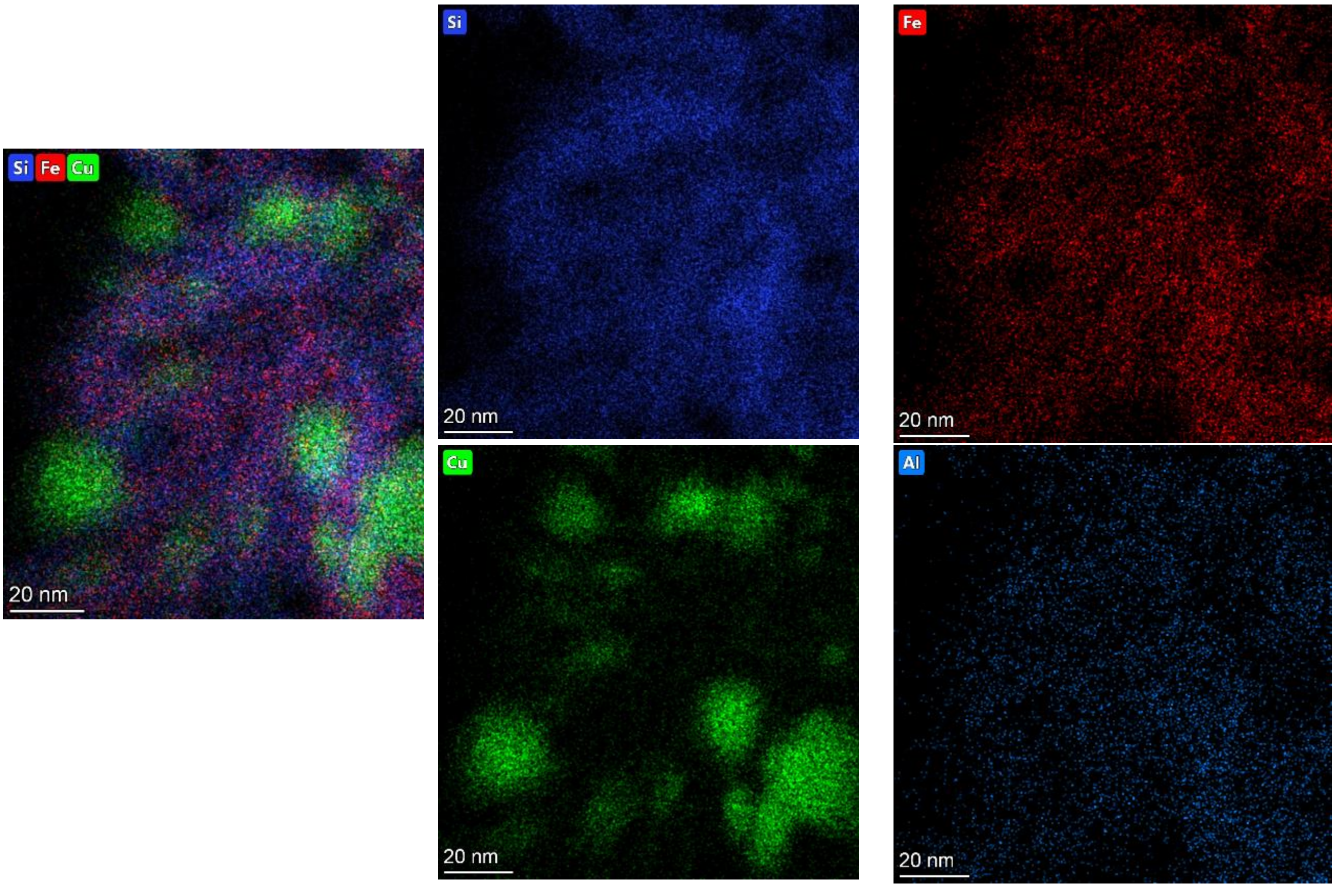

Figure S3. EDS elemental mapping images of fresh reduced and passivated 35Cu13Fe1Al-SiO_2 catalyst.
